# Buffalo Academy of Medicine

**Published:** 1902-04

**Authors:** F. W. McGuire

**Affiliations:** Secretary


### SOCIETY PROCEEDINGS.

### Buffalo Academy of Medicine.

*Stated Meeting, January 28, 1902.*

Reported by F. W. McGUIRE, M. D., Secretary.

THE stated meeting of the Buffalo Academy of Medicine was held at the Academy rooms, Public Library Building, Tuesday evening, January 28, 1902.

The meeting was called to order by the president, Dr. Charles G. Stockton, at 8.45 p.m. The minutes of the last stated meeting were read and approved.

The council recommended the election of Drs. Joseph B. Betz and Vertner Kenerson for resident membership. They were separately balloted for and elected fellows of the Academy.

The section on obstetrics and gynecology provided the following program:

Dr. William R. Pryor, professor of gynecology, New York, read a paper, entitled

THE SUPRAPUBIC AND VAGINAL METHODS OF TREATING PELVIC SUPPURATION. (See page 629.)

DISCUSSION.

Dr. M. D. Mann: In discussing the paper at some length said that in the main features he agreed with the speaker, but differed in some conditions. He agreed that all unilateral lesions should always be operated on through the abdomen. He also agreed that where pus was situated above pelvic brim, involving other adnexa, such as appendix, bowel and the like, the abdominal route should always be chosen.

When it came to discussing gonorrheal infection, however, Dr. Mann made some exceptions. In regard to conservative operations, where merely drainage was done early, he pointed out that these patients, while they were apparently physically well, were really not anatomically cured. He also said in double gonorrheal infections he thought the uterus and both tubes ought to come out, but said he preferred to do it through abdomen, believing he could do it better and in a shorter period of time by this route. Dr. Mann said he had given the subject of vaginal work a great deal of thought and was firmly convinced that certain conditions were preferably treated by vaginal method, but that he believed that the abdominal route had a much wider scope than was usually attributed to it by those accustomed to the vaginal route.

Dr. HERMAN E. HAYD eulogised the speaker for the very clear and able manner he presented his subject. He firmly agreed that pus in the pelvis should be treated as pus elsewhere. He thought the more acute the sepsis was the greater the indication for the vaginal route. He also spoke at some length on the complications necessarily arising in opening abdomen, such as adhesions, possibly leading later to intestinal obstruction and the like. Early vaginal incision in septic cases should be done in order to prevent the destruction of these organs.

Dr. C. C. FREDERICK made a plea for conservatism in removing all pelvic organs, as he said he had seen severe nervous conditions follow total ablation. He urged that where possible the ovary should not be removed ; he also thought that adhesion follows vaginal as well as the abdominal route.

Dr. S. Y. HOWELL believes it possible to save many organs by early vaginal drainage. He thinks where there is pus in pelvis the rational way seems to be through vagina. He believed both methods have their place and that each in certain conditions should be employed. He said the difficulty of vaginal work frequently led people to use abdominal route.

Dr. M. A. CROCKETT in a clear, concise way showed that the ability of a man to operate one way better than another should not be considered, but the question was which route should he choose in a given operation, the man having same skill in one as the other route. He thought that in all cases of general gonorrheal infection the uterus should be removed.

Dr. M. Hartwig thinks that each case is a law unto itself and it is difficult to lay down any set rule for all cases; he also thought we should be prepared to operate both ways.

Dr. William R. Pryor in closing the discussion said, we seem to be agreed on most of the points of the paper, but differ slightly in regard to the treatment of gonorrheal infections. He said he wished he knew of some agent which would destroy the gonococcus without injuring the tissues. He said the vaginal route was not a panacea for all pelvic operations.

On motion a vote of thanks was tendered Dr. W. R. Pryor for his interesting and instructive paper.

Adjourned 10 p.m. Attendance, 58.

